# Simultaneous whole-head electrophysiological recordings using EEG and OPM-MEG

**DOI:** 10.1162/imag_a_00179

**Published:** 2024-05-20

**Authors:** Zelekha A. Seedat, Kelly St. Pier, Niall Holmes, Molly Rea, Layla Al-Hilaly, Tim M. Tierney, Christine M. Embury, Rosemarie Pardington, Karen J. Mullinger, J. Helen Cross, Elena Boto, Matthew J. Brookes

**Affiliations:** Young Epilepsy, Surrey, United Kingdom; Sir Peter Mansfield Imaging Centre, School of Physics and Astronomy, University of Nottingham, University Park, Nottingham, United Kingdom; Cerca Magnetics Limited, Nottingham, UK; Wellcome Centre for Human Neuroimaging, UCL Queen Square Institute of Neurology, University College London, London, United Kingdom; Centre for Human Brain Health, School of Psychology, University of Birmingham, Edgbaston, Birmingham, United Kingdom; Developmental Neurosciences, University College London NIHR Great Ormond Street Institute of Child Health, London, United Kingdom

**Keywords:** optically pumped magnetometer, OPM, magnetoencephalography, MEG, OPM-MEG, electroencephalography, EEG, simultaneous EEG/OPM-MEG, electrophysiology

## Abstract

Electroencephalography (EEG) and magnetoencephalography (MEG) non-invasively measure human brain electrophysiology. They differ in nature; MEG offers better performance while EEG (a wearable platform) is more practical. They are also complementary, with studies showing that concurrent MEG/EEG provides advantages over either modality alone, and consequently clinical guidelines for MEG in epilepsy recommend simultaneous acquisition of MEG and EEG. In recent years, new instrumentation—the optically pumped magnetometer (OPM)—has had a significant impact on MEG, offering improved performance, lifespan compliance, and wearable MEG systems. Nevertheless, the ability to carry out simultaneous EEG/OPM-MEG remains critical. Here, we investigated whether simultaneous, wearable, whole-head EEG and OPM-MEG measurably degrades signal quality in either modality. We employed two tasks: a motor task known to modulate beta oscillations, and an eyes-open/closed task known to modulate alpha oscillations. In both, we characterised the performance of EEG alone, OPM-MEG alone, and concurrent EEG/OPM-MEG. Results show that the signal to noise ratio (SNR) of the beta response was similar, regardless of whether modalities were used individually or concurrently. Likewise, our alpha band recordings demonstrated that signal contrast was stable, regardless of the concurrent recording. We also demonstrate significant advantages of OPM-MEG; specifically, the OPM-MEG signal is less correlated across channels and less susceptible to interference from non-brain sources. Our results suggest that there are no barriers to simultaneous wearable EEG/OPM-MEG, and consequently this technique is ripe for neuroscientific and clinical adoption. This will be important in the clinic where simultaneous EEG and OPM-MEG recordings will facilitate better interpretation of OPM-MEG data in patients.

## 1 Introduction

Electroencephalography (EEG) (Berger, 1929) and magnetoencephalography (MEG) (Cohen, 1968) provide non-invasive characterisation of brain electrophysiology. EEG records changing electrical potential difference at the scalp caused by current flow through neural assemblies. MEG measures changing magnetic fields above the scalp generated by similar currents. Both modalities allow interrogation of brain function in health and disease and offer significant clinical utility—particularly in disorders like epilepsy where a change in the electrical activity underlies symptoms.

EEG and MEG differ in both practicality and performance (Baillet, 2017): EEG is “wearable” (meaning lightweight sensors are placed in electrical contact with the scalp and move freely with the head, allowing adaptability to any head shape/size and free movement during data acquisition). This makes it well tolerated and lifespan compliant. It is also readily available and low cost. However, the high electrical resistance of the skull reduces the amplitude of measured potentials and spatially distorts the signal topography, meaning sensitivity and spatial resolution are limited. EEG is also susceptible to interference from muscles (Whitham et al., 2007) which degrades data quality. Conversely, MEG is less sensitive to muscle artefacts (Boto et al., 2019;Muthukumaraswamy, 2013) and offers better spatial resolution than EEG (since magnetic fields pass through the skull with little distortion). However, conventional MEG systems rely on superconducting sensors to measure the neuromagnetic field (Hamalainen et al., 1993). Because such sensors require low temperatures, they are fixed in a rigid helmet with a gap between the sensors and the scalp for thermal insulation. Consequently, signal magnitude is limited by sensor proximity; a problem that is amplified in participants with small heads. Moreover, participants must remain still relative to the fixed sensor array, which makes MEG hard to deploy in many participants (e.g., infants). In summary, while MEG offers improved performance, EEG has practical advantages due to its adaptability and wearability.

Despite their differences, a large body of evidence shows that EEG and MEG are complementary. MEG is most sensitive to current flow oriented tangential to the scalp whereas EEG is most sensitive to radial currents. This means, theoretically, simultaneous measurement offers improved coverage. This is realised in practice; for example,Aydin et al. (2015)showed that, in epilepsy, simultaneous EEG/MEG allowed mapping of spike propagation that was not possible via MEG or EEG alone. Likewise,Yoshinaga et al. (2002)used dipole modelling to show that MEG and EEG provide information that was not obtainable with either modality alone. These demonstrations show the importance of combining the two methods, and clinical guidelines reflect this by recommending concurrent recording in epilepsy (Bagić et al., 2011). However, the fixed nature of MEG means that, in concurrent EEG/MEG, the significant practical advantages of EEG are lost, and participants must remain still in a cumbersome machine, ruling out naturalistic behaviour and reducing patient comfort.

In recent years, MEG has been revolutionised by the introduction of new magnetic field sensors (see e.g.,Brookes et al., 2022;Schofield et al., 2023;Tierney et al., 2019for reviews). Optically Pumped Magnetometers (OPMs) are small and lightweight sensors which measure magnetic fields with a sensitivity comparable to superconducting sensors, but without cryogenic cooling. Because OPMs can get closer to the scalp than cryogenic sensors, they detect a signal that is less spatially diffuse and higher amplitude, bringing improved sensitivity and spatial resolution (Borna et al., 2017;Boto et al., 2016;Iivanainen et al., 2017;Nugent et al., 2022). Moreover, OPMs can be mounted in a lightweight helmet and move with the head, meaning that (assuming background field is controlled (Holmes et al., 2018,^2019^;Mellor et al., 2022;Robinson et al., 2022)) the MEG signal can be measured as a person moves (Boto et al., 2018). Whole-head OPM-MEG systems are emerging (e.g.,Alem et al., 2023;Boto et al., 2021;Hill et al., 2020;Rea et al., 2022;Rhodes et al., 2023) and the clinical potential of OPM-MEG is also being shown (Vivekananda et al., 2020)—for example, OPM-MEG offers higher sensitivity (compared to conventional MEG) for detection of epileptic spikes in children (Feys et al., 2022); it can record patient data during a seizure (Feys et al., 2023;Hillebrand et al., 2023) and a recent case study reported that, even for a deep source (mesiotemporal cortex), OPM-MEG could measure ~60% of the epileptic discharges that were identified using invasive EEG (Feys et al., 2024). These initial studies indicate that OPM-MEG provides the performance advantages of MEG within a package that is similar in form to EEG—a wearable, motion robust helmet.

OPM-MEG now opens the opportunity for the combined use of EEG and MEG within a single wearable system. Such a system would adapt to head shape/size and allow free head movement during data acquisition. It would enable the additional information content from concurrent MEG/EEG recordings without the loss of the major advantages of EEG. Perhaps most significantly, simultaneous measurements would allow clinicians to reap the significant advantages that OPM-MEG brings (over conventional MEG and EEG) while maintaining the clinical standard (EEG). Two previous studies (Boto et al., 2019;Ru et al., 2022) already show that OPMs can operate in close proximity to EEG electrodes. However,Boto et al. (2019)had only two OPMs and it was not possible at that stage to attain whole-head coverage. Similarly,Ru et al. (2022)used an integrated MEG-EEG-fNIRS system, but OPMs were placed over right frontal and temporal lobes only (not whole-head coverage) and there was no assessment of the impact on MEG, EEG, or fNIRS data quality in the presence of the other recording modalities. Here, we expand on these studies by demonstrating the simultaneous use of whole-head (64 channel) EEG and whole-head (128 channel) OPM-MEG. In this study, we collected data in 12 individuals using 1) EEG alone, 2) OPM-MEG alone, and 3) concurrent EEG/OPM-MEG. We restrict ourselves to channel-level analyses (to avoid confounding effects due to, e.g., differences in source localisation strategies) and we quantitatively compute signal to noise ratio (SNR) to test the hypotheses that: 1) The presence of whole-head OPM-MEG does not significantly degrade the SNR of EEG recordings and 2) the presence of whole-head EEG does not significantly degrade the SNR of OPM-MEG recordings. Successful confirmation of these hypotheses would demonstrate the utility of concurrent EEG/OPM-MEG for future clinical (and neuroscientific) applications.

## 2 Materials and Methods

All data were acquired at Young Epilepsy (Lingfield, Surrey, UK). This research was approved by the University of Nottingham Medical School Research Ethics Committee. Study participants were 12 adults aged between 26 and 64 (mean age 41, 8 female) with no known neurological conditions. They all gave written informed consent for the study. The data and code used in this study are available on GitHub:https://github.com/ZSeedat/EEG_OPMMEG_HealthyAdultStudy_YoungEpilepsy

### 2.1 Hardware

The OPM-MEG device is a complete integrated system (Cerca Magnetics Ltd., Nottingham, UK), including a sensor array, helmet for sensor mounting, patient support, magnetic shielding, and field control. It also includes equipment for participant motion tracking, coregistration of sensor locations to brain anatomy, stimulus delivery, and data acquisition (including delineation of stimulus timing).

The OPM array comprised 64 3rd-generation QuSpin dual-axis zero field magnetometers (QuSpin Inc, Colorado, USA) which provide 128 independent measurements of field around the head (128 channels). The sensors are integrated into a single system to simultaneously measure field and can be mounted in a series of rigid helmets of varying size, to accommodate individuals of any age.

To achieve a magnetically “quiet” environment, the system is housed in a magnetically-shielded room (MSR) (Holmes et al., 2022) comprising multiple layers of high permeability/conductivity metal which reduce both static and time-varying magnetic fields. The room is equipped with degaussing coils and these were used to demagnetise the innermost metal layer, which results in a reduction of static field inside the MSR, beyond that afforded by the room itself. The room is also equipped with a set of 27 “window” coils embedded within the walls. Given a measured field in the room, these coils can be independently controlled such that they generate a summed magnetic field which opposes the measured field, enabling precise field nulling in the space occupied by the participant. This in turn ensures minimisation of magnetic artefact as a participant moves.

The system includes a PC for delivering stimuli to the participant and instrumentation to facilitate time-locking between the stimulus and measured data (i.e., a “triggering” system). It has cameras for tracking participant motion (OptiTrack, NaturalPoint Inc., Oregon, USA) (via monitoring movement of infrared retro-reflective markers) and equipment for coregistration of sensor positions to brain anatomy.

A 64-channel MEG-compatible EEG system (Brain Products GmbH, Munich, Germany) was used to acquire all EEG data. This system comprised an EEG-cap (with passive Ag/AgCl electrodes), signal pre-amplifiers, main-amplifiers, a power pack, a USB adaptor box, and a data acquisition laptop. The pre-amplifiers were placed 50 cm from the participant. The two main signal amplifiers and power pack were also inside the MSR approximately 1 m from the participant, and ribbon cables connected the EEG cap with the amplifiers. Optical fibres relayed the signal from the amplifiers out of the MSR through waveguides to the recording laptop. The EEG cap had no measurable residual magnetic field and has previously been used for EEG recordings in a conventional MEG system. The pre-amplifiers had a residual magnetic field of 700 nT at their surface falling to 50 nT at a distance of 10 cm; their residual field was indistinguishable from the typical background field in the MSR at a distance of 20 cm (measured using a Fluxgate Magnetometer; Fluxmaster, Stefan Meyer, Dinslaken, Germany). The main amplifiers and powerpack had a residual field of 70 µT at their surface, falling to 400 nT at 20 cm and 20 nT at 40 cm. At a distance of 50 cm, their residual field was also indistinguishable from the background field. We therefore expected minimal influence of static field from the EEG equipment.

The ground electrode was AFz, and the reference electrode was FCz. 63 channels of data were recorded from the scalp, and the 64th channel recorded the electrocardiogram (ECG). EEG electrodes were connected to the head with a conductive gel, and the impedances of all good electrodes were kept below 10 kΩ on all subjects. During all EEG recordings, the cap was set up according to anatomical landmarks (10-10 layout). For simultaneous EEG/OPM-MEG recordings, a thermally insulated aerogel cap (Outdoor Research, Seattle, USA) was placed on top of the EEG cap and the OPM-MEG helmet placed on top of that. Sensor positions were aligned between the EEG and OPM-MEG systems (FC1 in the OPM-MEG helmet was lined up with Fpz on the EEG cap, seeSupplementary Information). An elasticated chinstrap held the helmet in position on the head.

**Fig. 1.**
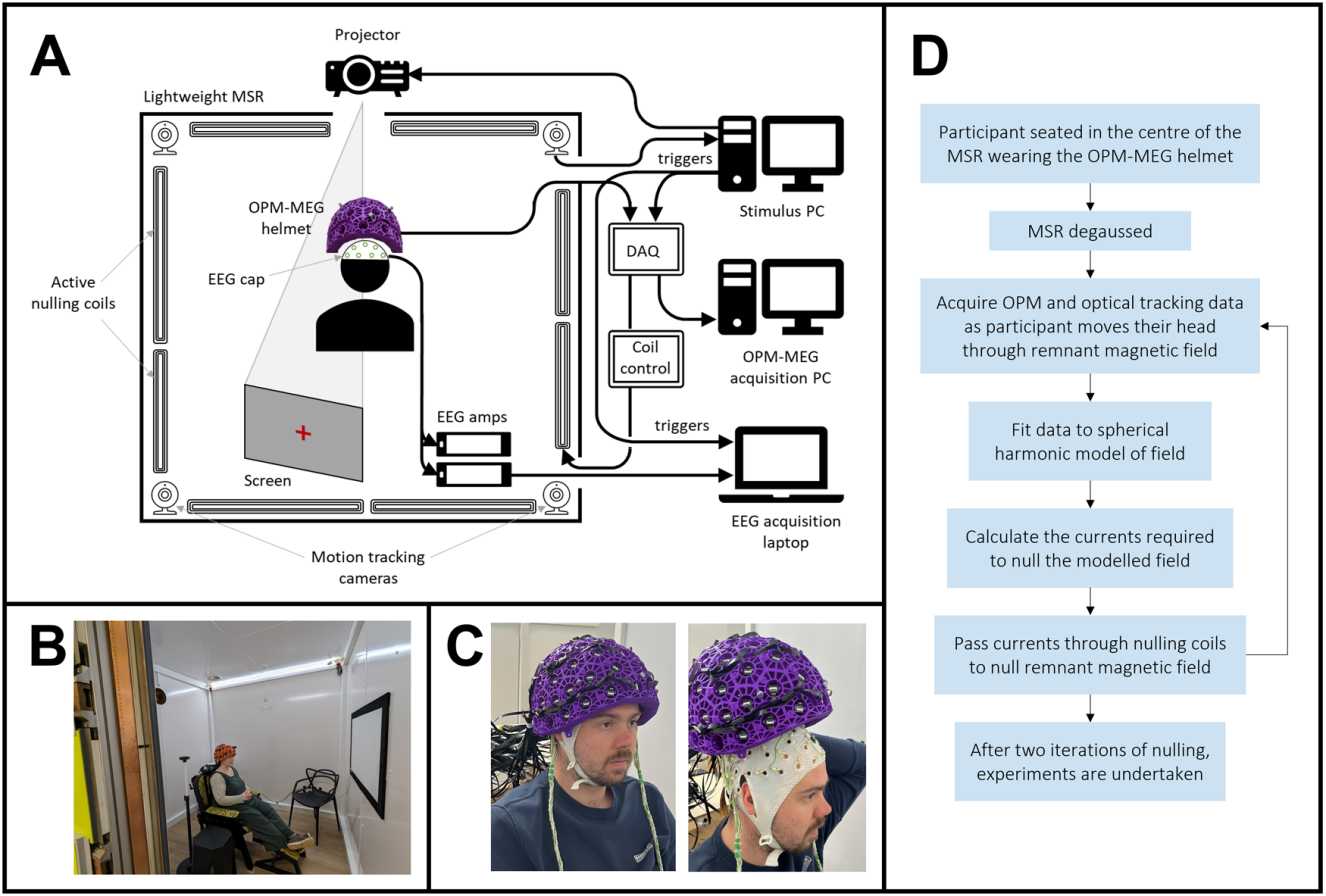
Experimental set-up and field nulling. (A) Schematic of the simultaneous OPM-MEG/EEG system. (B) The lightweight magnetically shielded room at Young Epilepsy where experiments took place. (C) Participants wore the OPM-MEG helmet (with or without an EEG cap). (D) Flow chart describing the field nulling process.

Participants were seated comfortably using a patient support, positioned at the centre of the MSR and a two-way intercom enabled communication between the experimenter and the participant.Figure 1Ashows a schematic representation of the system used.Figure 1Bis an outside view of the participant in the MSR, andFigure 1Cis a photo of the EEG and OPM-MEG helmets in place on a participant.

### 2.2 Experimental paradigms

Each participant underwent 3 experimental sessions: OPM-MEG only, EEG only, and simultaneous EEG/OPM-MEG. Each session took place in the MSR and lasted ~40 min. The order of sessions and tasks was pseudorandomised across participants but to avoid the practical limitations of washing conductive gel out of participants hair between sessions, the OPM-MEG only sessions were never placed between the EEG only and EEG/OPM-MEG sessions. All sessions were completed on the same day, and participants were offered a 5–10 min break between sessions during which time they were able to sit and relax. Within each session, participants performed 5 tasks; however, only 2 are reported:

1) **Motor task:**Participants wore a motion tracking marker on their right index finger and were asked to complete one brief finger abduction when a visual cue (an image of a hand) appeared on the screen. A single trial was 5 s in duration, and the visual cue was presented for 2 s. In the rest phase, a red fixation cross was shown. A total of 50 trials were recorded. This task elicits a robust change in beta (13–30 Hz) amplitude, including a movement-related beta decrease (MRBD) and a post-movement beta rebound (PMBR) (Pfurtscheller & Lopes da Silva, 1999).
2) **Alpha generation task:**Participants were asked to look at a red fixation cross on a grey screen. Every 30 s, they were asked by the experimenter to either close or open their eyes. This was repeated 5 times (i.e., 5 trials, each with duration 1 min). This is well known to drive increases or decreases in occipital alpha oscillations (Berger, 1929).

### 2.3 Nulling background fields

OPMs are vector field sensors, meaning if they move relative to a non-zero background field, they measure a signal that could obfuscate the neural signals of interest, and potentially stop the OPMs from working (Holmes et al., 2018). For this reason, nulling the field inside the MSR is important. Here, we used an approach originally described byRea et al. (2021). Briefly, the helmet was positioned on the participant’s head, the MSR door closed, and the room degaussed. The participant was then asked to execute a series of simple head movements. During these movements, we simultaneously recorded the magnetic field variations at the OPMs and head motion (via the motion tracking cameras). These synchronised data were input into a fitting algorithm to characterise the remnant field and its first-order gradient across the volume occupied by the helmet. The resulting field was used to determine coil currents which were then applied to the window coils. This generated a field equal and opposite to that measured, thereby cancelling it out. This procedure was carried out twice for each participant, followed by a third field map to measure the final fields in the room. This is summarised inFigure 1D.

### 2.4 Data acquisition

Field nulling—as described above—was carried out for experimental sessions where OPM-MEG was used (not for sessions where EEG was used alone). Total preparation time for EEG electrode application was approximately 40 min. OPM-MEG preparation time (including field nulling) was approximately 15 min.

For all experiments and sessions, OPM-MEG and EEG data were recorded at 1200 Hz and 1000 Hz respectively. Visual stimuli were shown via projection through a waveguide located behind the participant onto the wall of the MSR in front of the subject (visual angle 42° horizontally and 27° vertically). Motion tracking data (to monitor the finger abduction in the motor task) were captured simultaneously with OPM-MEG and EEG at a sample rate of 120 Hz. At the beginning of each experiment, a single synchronisation trigger was sent from the stimulus PC via a BNC tee adapter so that simultaneous markers appeared in the OPM-MEG and EEG data. Similar triggers were also sent to mark the beginning of each trial in the motor task. This enabled precise synchronisation of the OPM-MEG and EEG data. Data from peripheral recordings (e.g., motion tracking) also generated triggers sent directly to the OPM-MEG acquisition PC to enable data synchronisation.

### 2.5 Pre-processing

Pre-processing was performed in MATLAB using the FieldTrip toolbox (Oostenveld et al., 2011), and the same pipeline was used for EEG and OPM-MEG. Data were bandpass filtered between 1 and 150 Hz using a 4th-order Butterworth filter, notch filtered at 50 Hz and 100 Hz to remove mains frequency noise and its harmonic (also using a 4th-order Butterworth filter), baseline corrected, and detrended with a linear fit. Data were then segmented into trials for each task:

- Motor task trials were defined as 1 s before movement cessation (measured by the motion tracking cameras) to 4 s after movement cessation (i.e., 5-s total duration).
- Alpha trials were defined from 1 s after the instruction to open/close their eyes (to allow for slow reaction times) until 6 s after the instruction (i.e., 5-s trial duration). This time window was taken because participants who are alert with their eyes closed are known to elicit the highest amplitude alpha response at the start of the trial (Nunez, 2002) (towards the end of the eyes closed period they are likely to be in a drowsy state which would attenuate alpha).

The signal variance for each trial and channel was inspected visually, and outliers (i.e., those trials with obvious artefacts) were removed. Homogeneous field correction (to reduce the effects of external interference) was applied to OPM-MEG data (Tierney et al., 2021), and EEG data were re-referenced (to the average of all good channels for the motor task, and the average of all good channels excluding those over occipital areas (Pz, POz, Oz, P1-8, PO3, PO4, PO7-10, O1, O2) for the alpha task). To ensure that our results were robust to changes in the EEG referencing scheme, all EEG analyses were repeated using the common recording reference (CRR). These additional results are shown in theSupplementary Information.

For the motor task, 2 participants were excluded from further analyses because there was no task-modulated response in EEG or OPM-MEG. One further participant was excluded due to missing data (motion tracking and the EEG/OPM-MEG synchronisation trigger failed), and this left 9 participants. For the alpha generation task, no participants were excluded.Tables 1and2show the numbers of trials and channels left after outliers had been removed.

**Table 1.** The numbers of participants, good channels, and good trials after pre-processing for the motor task.

**Motor task**	**EEG only**	**MEG only**	**EEG with MEG**	**MEG with EEG**
No. of participants	9	9	9	9
No. of good channels (mean ± std)	57 ± 2	127.4 ± 0.9	57 ± 2	126 ± 1
No. of good trials (mean ± std)	48 ± 4	47 ± 2	48 ± 2	47 ± 2

**Table 2.** The numbers of participants, good channels, and good trials after pre-processing for the alpha generation task.

**Alpha generation**	**EEG only**	**MEG only**	**EEG with MEG**	**MEG with EEG**
No. of participants	12	12	12	12
No. of good channels (mean ± std)	59 ± 2	126 ± 2	58 ± 2	126 ± 1
No. of good trials (mean ± std)	5 ± 0	4.9 ± 0.3	5 ± 0	4.8 ± 0.4

### 2.6 Field nulling analysis

To estimate the efficacy of the field nulling, the root mean square (RMS) field, based on both the uniform field and first-order field gradients, was computed on a spherical surface enclosing the head, and the range of head movements (see alsoSupplementary Information). A sphere of radius 0.25 m was chosen based on the range of head movements measured during field mapping.

### 2.7 Motor task analysis

Following pre-processing, time-frequency spectrograms (TFSs) were computed for every OPM-MEG and EEG channel: briefly, data were bandpass filtered into 18 overlapping frequency bands using a series of 3rd-order Butterworth filters. The signal from each band was Hilbert transformed to compute the analytic signal, and the absolute value taken to give the envelope of oscillatory amplitude. Concatenation in the frequency dimension produced the TFS, which was averaged across trials. The TFS was baseline corrected by subtracting the mean amplitude in the 3–4 s time window (relative to movement offset). The TFS was then divided by the baseline, to give a measure of relative change.

For all channels (OPM-MEG and EEG) we estimated beta band signal to noise ratio (SNR). An MRBD window was defined as -1 s to 0 s, and a PMBR window defined as 0.5 s to 1.5 s (both relative to movement cessation). SNR was calculated as the difference in mean signal amplitude between the two windows, divided by the standard deviation of the signal in the MRBD window. SNR was calculated for all channels, and data from the channels with the highest SNR were averaged across participants. We tested for statistically significant differences: that is, if OPM-MEG SNR was changed by the presence of EEG, and if EEG SNR was changed by OPM-MEG. This was assessed using a Wilcoxon rank-sum test.

In addition to SNR, we derived a measure of how far the signal from motor cortex spreads across scalp electrodes/sensors (as a result of volume conduction (EEG) or magnetic field spread (OPM-MEG)). For both EEG and OPM-MEG data, we measured Pearson correlation (with zero temporal lag) between the signal from the channel with the highest SNR, and all other channels (signals were beta envelopes, unaveraged across trials). We then counted the number of channels with a correlation above 0.3 and designated them “highly correlated.” The proportion of highly correlated channels was then calculated for each participant and averaged. This was carried out independently for EEG and OPM-MEG.

### 2.8 Alpha generation task analyses

Visual inspection of data from the alpha task was undertaken using AnyWave (Colombet et al., 2015): first independent component analysis (ICA) was used to remove the heartbeat artefact (using the AnyWave infomax implementation of ICA). Data were then bandpass filtered between 2 and 40 Hz, and notch filtered at 50 Hz (again using AnyWave). The filtered EEG and OPM-MEG data were inspected visually to look for the presence of alpha oscillations.

Spectral analysis was also undertaken: data were frequency filtered in the 1–150 Hz band using a 4th-order Butterworth filter. Data were then segmented into trials, and power spectral density (PSD) in the eyes-open and eyes-closed windows was estimated using the MATLAB periodogram function. We then computed a measure of signal contrast, defined as 8–13 Hz spectral power with eyes closed, divided by eyes open. The channel with the highest contrast was selected for each participant, and we averaged PSDs across individuals. We then tested for differences in contrast between sessions, with or without simultaneous recordings.

### 2.9 Head motion analysis

As a final analysis, the effects of head motion on EEG and OPM-MEG data were investigated for the motor task data. Motion tracking data were used to determine the position of the helmet throughout the recording. These position values were then differentiated to estimate helmet velocity as a function of time. The magnitude of this was calculated to give a single value of speed of helmet movement at each time point. “High velocity” time points were identified by thresholding at a value of 0.1 ms^-1^(this value was determined empirically by visual inspection). Where this threshold was exceeded, a marker was placed in the data and a head motion “trial” defined, beginning 2 s before the marker and ending 3 s after the marker.

Pre-processed EEG and OPM-MEG data were segmented according to the head-motion-trial markers and TFSs calculated in the 1–130 Hz frequency band, as described above. For each trial, the TFS was baseline corrected by subtracting the mean amplitude of the signal in the -2 s to -1 s window relative to the marker. The TFS was then divided by the baseline, to give a measure of relative change. The average TFS across trials and subjects was calculated for each OPM-MEG and EEG channel.

## 3 Results

### 3.1 Nulling background fields

Field nulling experiments were completed successfully in 11 out of 12 participants, with technical difficulty on the day of scanning preventing measurement in one person. Results are shown inFigure 2; before nulling, the RMS field over a spherical volume, with and without EEG, was 4.5 nT ± 0.6 nT and 5.0 nT ± 0.5 nT (mean ± standard deviation across participants) respectively. This dropped to 0.7 nT ± 0.5 nT and 0.7 nT ± 0.3 nT (with and without EEG, respectively) after field nulling. A Wilcoxon rank-sum test suggested no significant difference in the efficacy of nulling (p = 0.5), with and without EEG.

**Fig. 2.**
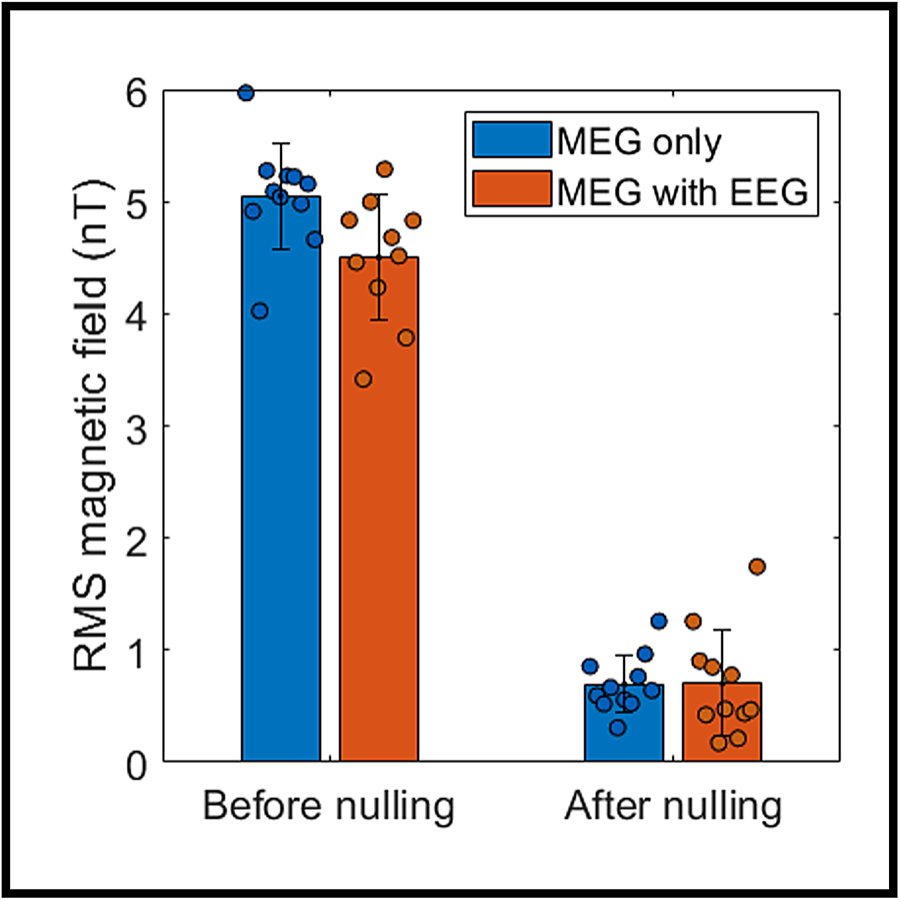
EEG does not significantly affect the efficacy of the field nulling process. Data points show the RMS field for individual sessions before and after nulling, bars show the mean, and error bars show the standard deviation. Blue shows the data for OPM-MEG only, and the orange bars are for OPM-MEG in the presence of EEG.

### 3.2 Motor task

**Fig. 3.**
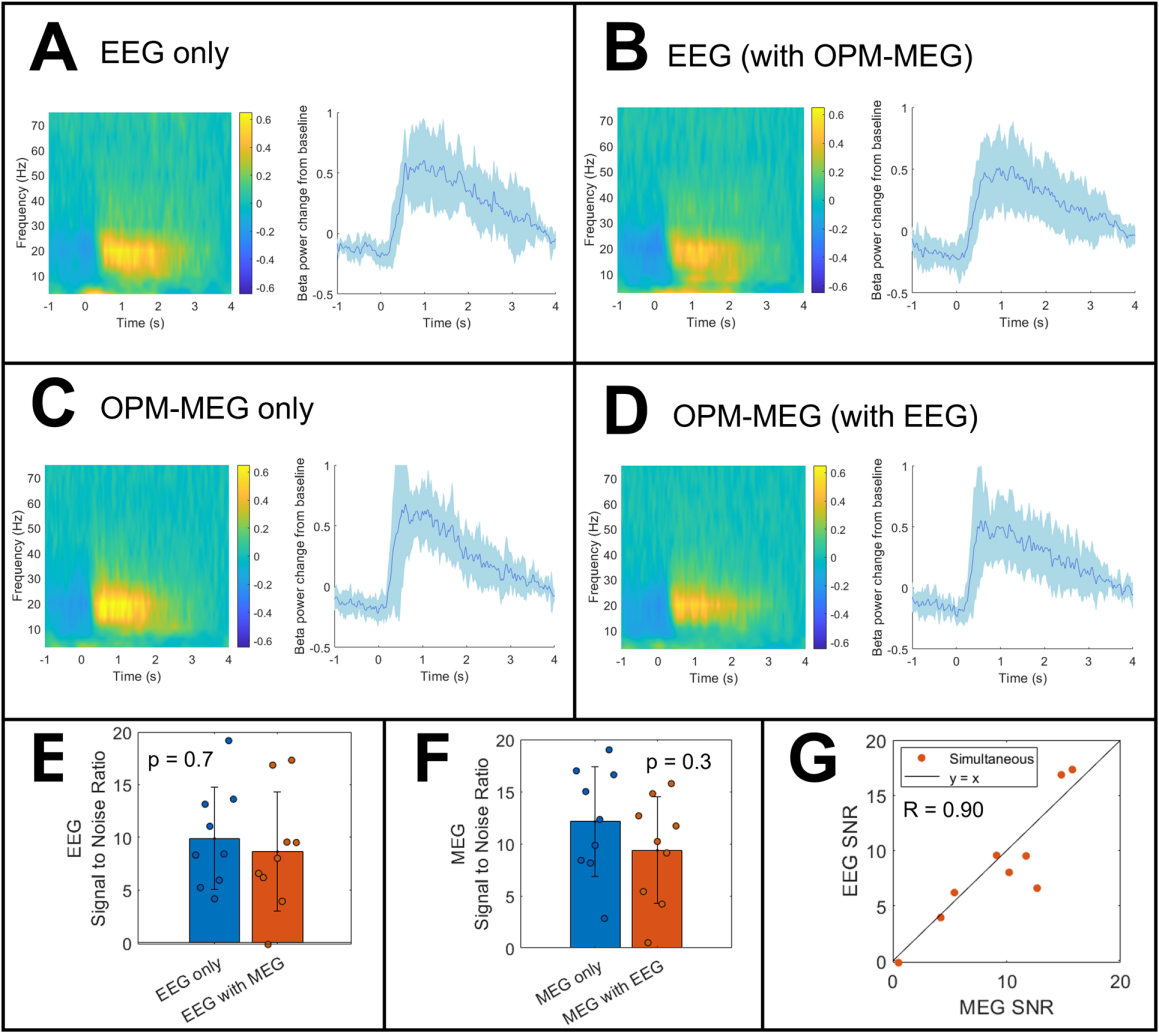
Finger abduction results. Panels (A and B) show the average TFS (left) and beta band envelope (right) in the channel with the highest SNR for EEG alone and EEG in the presence of OPM-MEG, respectively. In both plots, a time of 0 s indicates the offset of finger movement. Panels (C and D) show equivalent responses for OPM-MEG only and OPM-MEG in the presence of EEG, respectively. Panel (E) shows SNR values for EEG, and panel (F) shows equivalent values of OPM-MEG. Each data point represents a participant; the bars represent the mean over participants, and the error bars describe standard deviation. Panel (G) plots EEG SNR against OPM-MEG SNR, for the simultaneously recorded data; again, a single data point represents an individual participant, and the solid black line represents y = x.

Table 1shows the number of participants, trials, and channels remaining in each dataset after preprocessing.Figure 3, panels A–D, show TFSs and beta band oscillatory envelopes for the finger abduction task. In all cases, data have been averaged across participants and, in the case of the line plots, the shaded area represents standard deviation across participants. Data from channels with the highest SNR are shown. EEG data, with and without OPM-MEG, are shown in panels A and B respectively. OPM-MEG data, with and without EEG, are shown in panels C and D.

In all cases, the MRBD and PMBR are clearly visible and, most importantly, the addition of the simultaneous recording appears to have little effect on the result. SNR values for the peak channels are shown inFigures 3E(for EEG) and 3 F (for OPM-MEG). Considering EEG, the SNR when used alone was 10 ± 5 (mean ± std) and dropped marginally to 9 ± 6 when OPM-MEG was added, but this was not significant (p = 0.7; Wilcoxon rank-sum test). For OPM-MEG, when used alone, the SNR was 12 ± 5 which dropped to 9 ± 5 when the EEG was added, but again this was a non-significant effect (p = 0.3; Wilcoxon rank-sum test).Figure 3Gshows the SNR of OPM-MEG plotted against the SNR of EEG for simultaneously acquired data; as expected, participants with low SNR in OPM-MEG also have low SNRs in EEG (Pearson correlation 0.9; p = 0.001).

InFigure 4, panels A and B show TFS data as a function of channel location for a single representative participant (for OPM-MEG, only the radially oriented channels are shown for ease of visualisation). Note that, qualitatively, the signal from the motor cortex appears to impact more scalp locations in EEG than it does for OPM-MEG. This is formalised inFigure 4Cwhere the proportion of channels that are highly correlated with the peak response is shown for the two modalities. A Wilcoxon rank-sum test showed that there were significantly fewer highly correlated channels (p = 4 x 10^-5^) in OPM-MEG compared to EEG. This is an important finding and will be discussed further below.

**Fig. 4.**
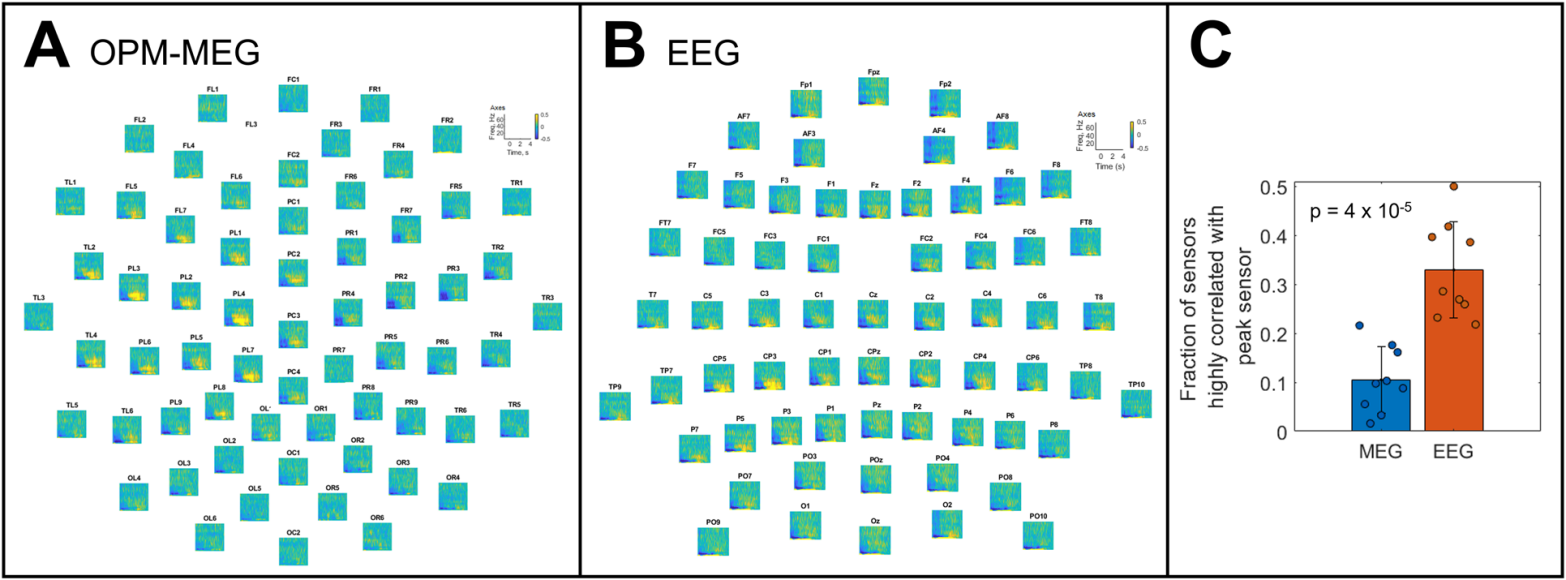
Volume conduction and field spread: Panels (A and B) show the time-frequency spectrograms across all sensors, for simultaneous OPM-MEG (left) and EEG (right) in a single representative participant. The EEG has been re-referenced to the average of all sensors. There is visibly greater signal spread across the scalp in EEG compared with OPM-MEG, as would be expected. Signal spread is quantified in panel (C) where the fraction of sensors that are highly correlated (R > 0.3) with the peak sensor is plotted. Data points show individuals, the bars represent the mean value, and error bars represent standard deviation. The fraction of highly correlated sensors is significantly lower in OPM-MEG compared with EEG.

### 3.3 Alpha generation task

Table 2shows the number of participants, trials, and channels remaining in each dataset after preprocessing for the alpha generation task.

**Fig. 5.**
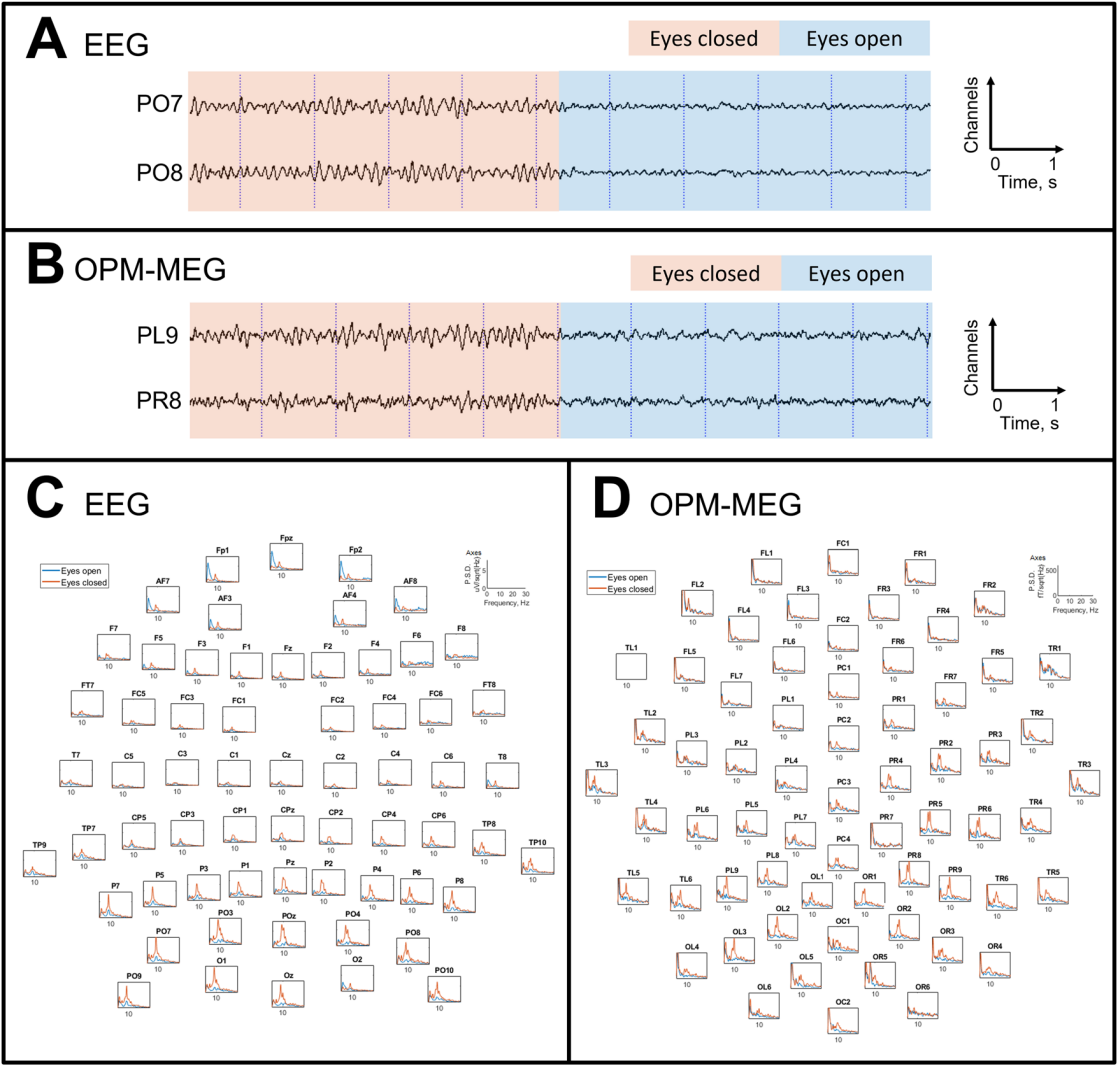
Alpha activity generated by eye closure in a representative participant, for simultaneous EEG and OPM-MEG. Panels (A and B) show simultaneous EEG and OPM-MEG data, filtered between 2 and 40 Hz. Panels (C and D) show the PSD plots for each channel across the whole head for EEG and OPM-MEG respectively. There is a clear alpha peak (~10 Hz) during the eyes-closed period which dominates posterior channels, as expected.

Figure 5shows, in a single representative participant, the increase in alpha activity when the eyes are closed. The time courses inFigure 5A and Bshow simultaneously acquired EEG and OPM-MEG recordings over a 10 s period (5-s eyes closed; 5-s eyes open). Alpha oscillations are clear in both modalities and are reduced when the eyes are opened. A spectral analysis for this participant is shown inFigure 5C and D, for all channels in EEG (panel C), and the radial channels in OPM-MEG (panel D). PSDs are plotted for the eyes-open segments (blue) and eyes-closed segments (orange) with a clear peak at ~10 Hz in the posterior channels, when eyes were closed.

**Fig. 6.**
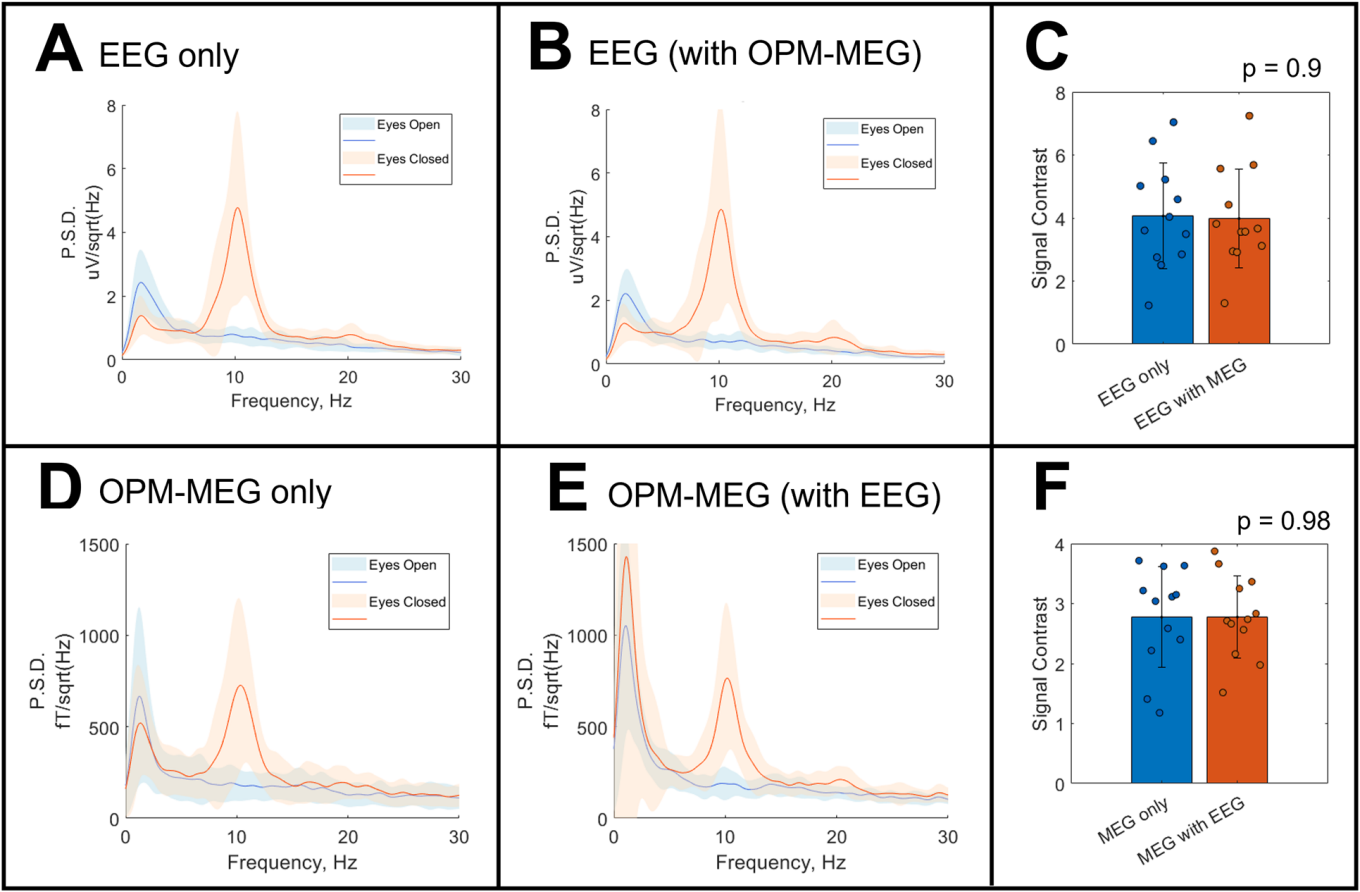
Spectral analysis of the alpha generation task. Panels (A and B) show the PSD plots from the best channels for EEG alone and EEG in the presence of OPM-MEG respectively. The solid line is the mean over participants, and the shaded area represents standard deviation. The EEG signal contrast values are shown in panel (C): data points represent single individuals; the bar shows the mean and the error bar standard deviation. Panels (D and E) show the PSDs for OPM-MEG alone and in the presence of EEG respectively. Panel (F) shows the signal contrast values.

The channels with the highest signal contrast (between eyes open and closed) were extracted for all participants and the participant-averaged PSDs are shown inFigure 6; eyes open in blue, eyes closed in orange.Figure 6A and Bshow EEG only and EEG recorded in the presence of OPM-MEG.Figure 6D and Eshow OPM-MEG only and OPM-MEG recorded in the presence of EEG. Again, the presence of the additional modality appears to have little effect on the alpha band signal contrast (though the OPM-MEG signal at very low (<5 Hz) frequency does increase significantly (p = 0.02 with eyes closed, p = 0.04 with eyes open) with the presence of EEG).Figure 6C and Fformalise this finding by showing signal contrast values for EEG (C) and OPM-MEG (F). A Wilcoxon rank-sum test suggested that OPM-MEG has no effect on signal contrast in EEG (p = 0.9) and likewise that EEG has no effect on signal contrast in OPM-MEG (p = 0.98). This, in agreement with the results inFigure 3, suggests that concurrent OPM-MEG/EEG is feasible.

### 3.4 Head motion

**Fig. 7.**
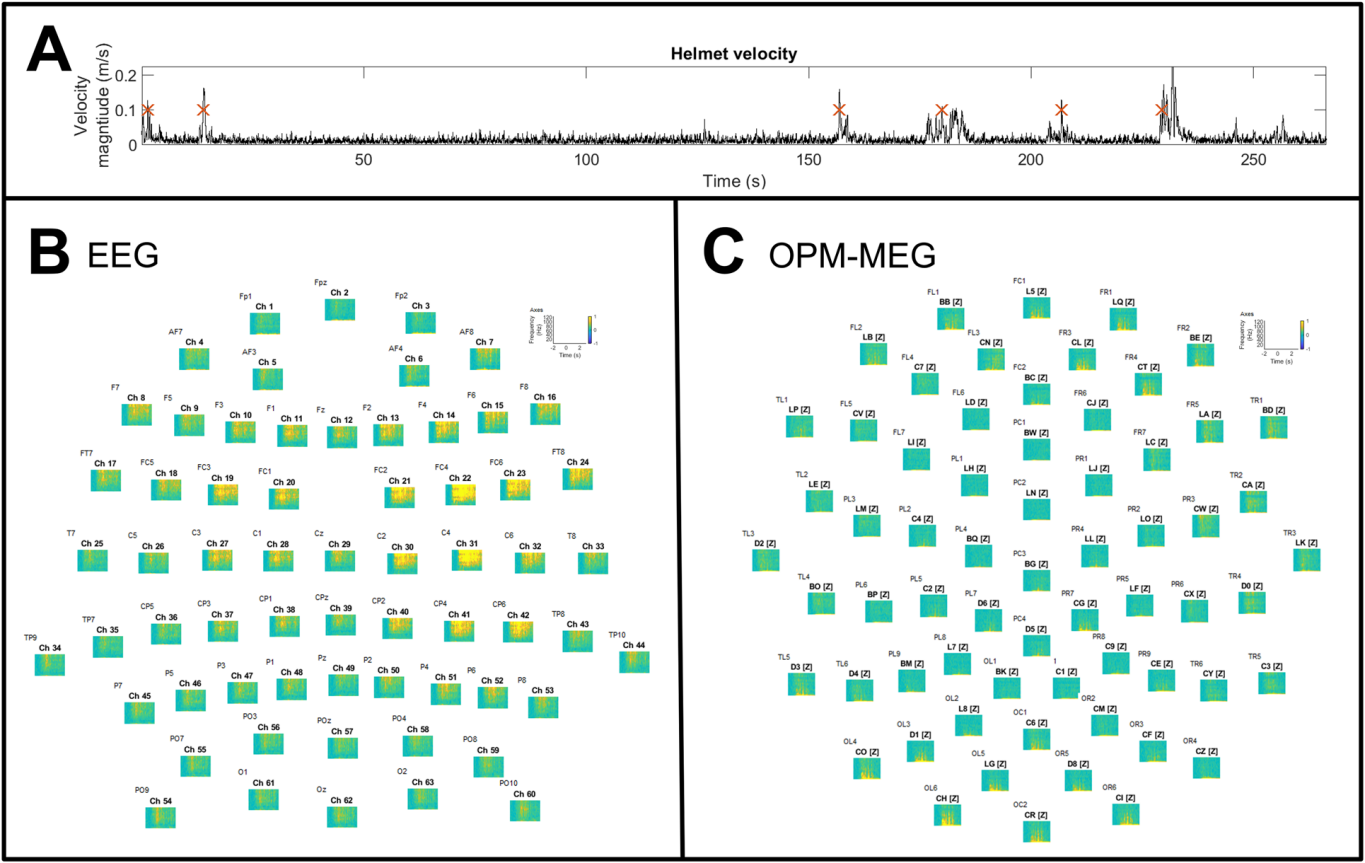
The effects of head motion on simultaneous EEG and OPM-MEG data. Panel (A) shows an example of the helmet speed measurement; where helmet speed exceeded 0.1 ms^-1^, a head motion trial was defined. In this participant, 6 trials were identified (delineated by the red crosses). Panels (B and C) show the trial and subject averaged TFSs in the 1–130 Hz frequency range for EEG and OPM-MEG respectively. There is marked high-frequency contamination in the EEG which is less prominent in the OPM-MEG data.

In total, we identified 112 head motion trials (where head velocity exceeded a threshold value of 10 cms^-1^) across all 12 subjects (the precise number of trials varied for each subject depending on how much they moved, as would be expected).Figure 7shows the effects of head motion on EEG and OPM-MEG data recorded during the motor task. The EEG data show marked high-frequency artefacts in centroparietal, temporal and some frontal electrodes, likely caused by electrophysiological activity in the muscles. In contrast, the OPM-MEG data have little high-frequency muscle artefacts, though some low-frequency artefact, likely caused by the movement of the sensors through a non-zero magnetic field, is measurable. These effects will be addressed further in our discussion below.

## 4 Discussion

The aim of this study was to show that simultaneous, wearable, whole-head EEG and OPM-MEG is feasible. To this end, we have acquired data across two tasks: a motor task known to modulate beta oscillations, and an eyes-open/closed task known to modulate alpha oscillations. In the former, the SNR of the beta response was similar, regardless of whether modalities were used individually or concurrently. Likewise in the latter experiment, signal contrast was stable regardless of the concurrent recording. The amplitude of the background magnetic field inside the room (a critical consideration to enable free participant movement in OPM-MEG) was also unchanged by the presence of EEG. These results suggest that simultaneous wearable EEG/OPM-MEG is possible.

The utility of simultaneous EEG and conventional MEG has been well documented: the high sensitivity to tangential sources in MEG, and the high sensitivity to radial sources in EEG, are complementary and from a theoretical point of view this improves uniformity of coverage and sensitivity. Moreover, from a clinical perspective, multiple studies have demonstrated that simultaneous recordings offer non-redundant information, and this is reflected in clinical practice guidelines (set out by the American Clinical Magnetoencephalography Society (ACMEGS)) which state that standard scalp EEG should be recorded simultaneously with MEG for assessment of patients with epilepsy (Bagić et al., 2011). This is not only advantageous due to non-redundant information but is also useful to grow confidence in MEG: EEG has been used for many years for the diagnosis and management of disorders, including epilepsy, tumours, dementia, head injury (including concussion), and encephalitis, whereas the high cost and limited practicality of conventional MEG have led to an under-utilisation (Bagić et al., 2023). Concurrent recording means that clinicians who are less familiar with MEG can keep the widely established EEG measures while simultaneously gaining new information from MEG. The usability of a wearable platform, coupled with the high spatial resolution and sensitivity afforded by OPM-MEG (compared to conventional MEG) (Boto et al., 2019;Hill et al., 2020;Rhodes et al., 2023), mean that concurrent OPM-MEG and EEG is even more attractive than the fusion of EEG with conventional MEG. For these reasons, the findings presented in this paper are important.

Although there was no measurable difference in SNR between OPM-MEG data acquired with and without EEG, there is a small reduction in mean OPM-MEG SNR, when simultaneous EEG was added (seeFig. 3F) and this warrants consideration. For the measurements acquired, we used two independent systems and so the presence of the EEG electrodes necessarily moved the OPMs slightly further from the scalp (approximately 8 mm from the scalp to the sensor casing compared with approximately 5 mm for OPM-MEG alone). Since the OPM-MEG signal amplitude drops with the square of the distance from the source, even the relatively low thickness of an EEG electrode and cap (~3 mm) will move the OPMs sufficiently to reduce signal strength noticeably. This was masked by inter-individual differences in the present data, and so the drop in amplitude was not significant. Nevertheless, the presence of EEG will (marginally) reduce the amplitude of the OPM-MEG signal and, for this reason, future systems might aim to accommodate the EEG electrodes within the OPM-MEG helmet. This should be possible, particularly if new generations of OPM-MEG system involve flexible (EEG-cap-like) helmets.

An important result in this paper is the finding inFigure 4that the proportion of “highly correlated” sensors was larger in EEG than in OPM-MEG. In EEG, electrical potentials are “smeared out” across the scalp by a combination of volume conduction, and the high resistivity of the skull. The former means that a single source in the brain affects the signal at multiple sensors, whereas the latter makes this process hard to mathematically model (Baillet, 2017). The result is that, as shown inFigure 4B, the signal (in this case from motor cortex) is spread to a large number of channels and this, coupled with the difficulty in modelling, leads to EEG having limited spatial resolution. In MEG, the magnetic field propagates from a single source to multiple sensors, resulting in a similar problem (though the relative transparency of the skull to magnetic field makes the MEG forward problem more tractable, and consequently gives MEG a better spatial resolution). The extent of the field spread depends on proximity of the sensors to the scalp and leads to more diffuse signals in conventional MEG (where cryogenic sensors are necessarily more distal) than OPM-MEG (where sensors are closer to the scalp surface). It is known that the ability to disentangle two sources in the brain depends strongly on how much the fields correlate at the sensor level (Boto et al., 2019;Brookes et al., 2021;Hill et al., 2024), and therefore the more the field spreads (i.e., the higher the proportion of highly correlated sensors across the scalp) the lower the ultimate spatial resolution of the technique will be. Our analysis therefore demonstrates directly the significant advantage of OPM-MEG over EEG in terms of spatial specificity—we observe a significantly more focal field pattern.

The advantages of MEG over EEG in terms of spatial accuracy are well documented, however a less well-known advantage is that MEG data are less susceptible to artefacts from non-neuronal sources (particularly muscle artefacts) compared with EEG (Boto et al., 2019;Muthukumaraswamy, 2013). This was demonstrated here by assessing time-frequency signatures of data from both modalities during periods of head movement. EEG data showed the expected high-frequency artefacts that are characteristic of muscle activity, whereas the OPM-MEG data were less susceptible to such activity. This is an important finding since it shows that, in a wearable system where movement is not only allowed, but in some experiments (e.g., naturalistic neuroscientific paradigms) could be encouraged, OPM-MEG offers significant advantages, particularly when measuring activity in the higher (beta and gamma) frequency ranges. This said, using OPM-MEG there remains some movement artefact at low frequency; this is due to movement of the sensors through the static magnetic field. Indeed, it is noteworthy that the most affected sensors were over frontal and posterior channels, and this orientation (anterior-posterior) is the one with the largest field gradient in the MSR used. Related to this, the result shown inFigure 6Esuggests that the presence of EEG enhances low-frequency OPM-MEG signal. Initially, this was thought to be related to possible increased movement when wearing both modalities; however, motion tracking data showed that there was no significant difference in maximum movement during the scan between participants wearing only the OPM-MEG helmet (30 ± 20 mm), or wearing simultaneous EEG/OPM-MEG (24 ± 17 mm) (mean ± std) for this task. It is therefore likely that the increased low-frequency signal was caused by increased high spatial frequency (non-linear) static fields inside the MSR due to the presence of the EEG amplifiers. Such fields were not modelled by our field nulling procedure, but the resulting artefacts could be suppressed in post-processing (indeed, there are several ways to do this, and multi-axis OPM design is of considerable benefit in this regard (Brookes et al., 2021)). Nevertheless, this finding demonstrates the critical need to drive background static fields inside an MSR as low as possible, using active magnetic shielding. This should be the topic of future study.

There are some limitations of the present study that should not be overlooked. Firstly, our findings relate to only two experimental paradigms, modulation of beta oscillations by finger movement and modulation of alpha oscillations by opening and closing the eyes. There are other signals that could have been measured (e.g., evoked responses). It seems likely that the present findings will extend to these other signals, however this may not necessarily be the case (e.g., for high frequencies, EEG signals are obfuscated by fields from muscle activity (Boto et al., 2019;Muthukumaraswamy, 2013)). Thus, future studies should aim to characterise fully the similarities and differences between EEG and OPM-MEG data acquired simultaneously across the whole frequency spectrum from delta through to high gamma bands. Second, a key limitation of EEG is that it measures the difference in electric potential between each scalp electrode and a reference, meaning results can change depending on where the reference is chosen. For the results presented here, we chose an average reference so that the EEG was not reliant on a single scalp electrode. To ensure that our results were robust, we repeated the analyses with the common recording reference (CRR, electrode FCz) and results are shown inSupplementary Information. We found that the impact of changing the referencing system was minimal; nevertheless, this choice of reference is likely to affect further signal analyses (e.g., source localisation). For our signal spread analyses, the number of channels was different for EEG (63 excluding ECG) and OPM-MEG (128), and OPM-MEG channels were recorded in two different orientations (radial and tangential with respect to the scalp). This makes a direct comparison between modalities difficult, as does the distance between adjacent sensors. However, the distance between adjacent EEG and OPM-MEG sensors was similar (~4 cm) with roughly even coverage of 63 and 64 sensors across the scalp respectively. Finally, here we have deliberately only employed channel-space analysis; we do not propose that this is the best way to present MEG data, rather we made this choice to enable a direct testing of our hypotheses and to avoid confounds associated with the relative advantages and disadvantages of source reconstruction methodologies (which differ between modalities). Had we chosen to undertake source reconstruction, this would likely improve the signal to noise of both OPM-MEG and EEG, but the fundamental finding (that both EEG and OPM-MEG work irrespective of the other modality) would remain.

## 5 Conclusion

In this study, we successfully acquired simultaneous whole-head EEG and OPM-MEG data in 12 healthy adults. Our results show that there is no statistically significant difference in OPM-MEG signal quality with or without the presence of EEG and vice versa. This indicates that future clinical or research studies can employ simultaneous EEG and OPM-MEG (with appropriate MEG-compatible equipment) without significantly affecting data quality.

## Supplementary Material

Supplementary Material

## Data Availability

The data and code used in this study are available on GitHub:https://github.com/ZSeedat/EEG_OPMMEG_HealthyAdultStudy_YoungEpilepsy.

## References

[b1] Alem , O. , Hughes , K. , Buard , I. , Cheung , T. , Maydew , T. , Griesshammer , A. , Holloway , K. , Park , A. , Lechuga , V. , Coolidge , C. , Gerginov , M. , Quigg , E. , Seames , A. , Kronberg , E. , Teale , P. , & Knappe , S. ( 2023 ). An integrated full-head OPM-MEG system based on 128 zero-field sensors . Frontiers in Neuroscience , 17 , 1190310 . 10.3389/fnins.2023.1190310 37389367 PMC10303922

[b2] Aydin , Ü. , Vorwerk , J. , Dümpelmann , M. , Küpper , P. , Kugel , H. , Heers , M. , Wellmer , J. , Kellinghaus , C. , Haueisen , J. , Rampp , S. , Stefan , H. , & Wolters , C. ( 2015 ). Combined EEG/MEG can outperform single modality EEG or MEG source reconstruction in presurgical epilepsy diagnosis . PLoS One , 10 ( 3 ), e0118753 . 10.1371/journal.pone.0118753 25761059 PMC4356563

[b3] Bagić , A. , Bowyer , S. , Burgess , R. , Funke , M. , Lowden , A. , Mohamed , I. , Wilson , T. , Zhang , W. , Zillgitt , A. , & Tenney , J. ( 2023 ). Role of optically pumped magnetometers in presurgical epilepsy evaluation: Commentary of the American Clinical Magnetoencephalography Society . Epilepsia , 64 ( 12 ), 3155 – 3159 . 10.1111/epi.17770 37728519

[b4] Bagić , A. , Knowlton , R. , Rose , D. , & Ebersole , J. ( 2011 ). American Clinical Magnetoencephalography Society Clinical Practice Guideline 1: Recording and analysis of spontaneous cerebral activity . Journal of Clinical Neurophysiology , 28 , 348 – 354 . https://journals.lww.com/clinicalneurophys/fulltext/2011/08000/american_clinical_magnetoencephalography_society.4.aspx# 21811121 10.1097/WNP.0b013e3182272fed

[b5] Baillet , S. ( 2017 ). Magnetoencephalography for brain electrophysiology and imaging . Nature Neuroscience , 20 , 327 – 339 . 10.1038/nn.4504 28230841

[b6] Berger , H. ( 1929 ). Über das Elektrenkephalogramm des Menschen . Archiv für Psychiatrie und Nervenkrankheiten , 87 , 527 – 570 . 10.1007/bf01797193

[b7] Borna , A. , Carter , T. , Goldberg , J. , Colombo , A. , Jau , Y. , Berry , C. , McKay , J. , Stephen , J. , Weisend , M. , & Schwindt , P. ( 2017 ). A 20-channel magnetoencephalography system based on optically pumped magnetometers . Physics in Medicine and Biology , 62 , 8909 . 10.1088/1361-6560/aa93d1 29035875 PMC5890515

[b8] Boto , E. , Bowtell , R. , Krüger , P. , Fromhold , M. , Morris , P. , Meyer , S. , Barnes , G. , & Brookes , M. ( 2016 ). On the potential of a new generation of magnetometers for MEG: A beamformer simulation study . PLoS One , 11 ( 8 ), e0157655 . 10.1371/journal.pone.0157655 27564416 PMC5001648

[b9] Boto , E. , Hill , R. , Rea , M. , Holmes , N. , Seedat , Z. , Leggett , J. , Shah , V. , Osborne , J. , Bowtell , R. , & Brookes , M. ( 2021 ). Measuring functional connectivity with wearable MEG . NeuroImage , 230 , 117815 . 10.1016/j.neuroimage.2021.117815 33524584 PMC8216250

[b10] Boto , E. , Holmes , N. , Leggett , J. , Roberts , G. , Shah , V. , Meyer , S. , Muñoz , L. , Mullinger , K. , Tierney , T. , Bestmann , S. , Barnes , G. , Bowtell , R. , & Brookes , M. ( 2018 ). Moving magnetoencephalography towards real-world applications with a wearable system . Nature , 555 , 657 – 661 . 10.1038/nature26147 29562238 PMC6063354

[b11] Boto , E. , Seedat , Z. , Holmes , N. , Leggett , J. , Hill , R. , Roberts , G. , Shah , V. , Fromhold , T. , Mullinger , K. , Tierney , T. , Barnes , G. , Bowtell , R. , & Brookes , M. ( 2019 ). Wearable neuroimaging: Combining and contrasting magnetoencephalography and electroencephalography . NeuroImage , 201 , 116099 . 10.1016/j.neuroimage.2019.116099 31419612 PMC8235152

[b12] Brookes , M. , Boto , E. , Rea , M. , Shah , V. , Osborne , J. , Holmes , N. , Hill , R. , Leggett , J. , Rhodes , N. , & Bowtell , R. ( 2021 ). Theoretical advantages of a triaxial optically pumped magnetometer magnetoencephalography system . NeuroImage , 236 , 118025 . 10.1016/j.neuroimage.2021.118025 33838266 PMC8249355

[b13] Brookes , M. , Leggett , J. , Rea , M. , Hill , R. , Holmes , N. , Boto , E. , & Bowtell , R. ( 2022 ). Magnetoencephalography with optically pumped magnetometers (OPM-MEG): The next generation of functional neuroimaging . Trends in Neurosciences , 45 ( 8 ), 621 – 634 . 10.1016/j.tins.2022.05.008 35779970 PMC10465236

[b14] Cohen , D. ( 1968 ). Magnetoencephalography: Evidence of magnetic fields produced by alpha-rhythm currents . Science , 161 ( 3834 ), 784 – 786 . 10.1126/science.161.3843.784 5663803

[b15] Colombet , B. , Woodman , M. , Badier , J. , & Bénar , C. ( 2015 ). AnyWave: A cross-platform and modular software for visualizing and processing electrophysiological signals . Journal of Neuroscience Methods , 242 , 118 – 126 . 10.1016/j.jneumeth.2015.01.017 25614386

[b16] Feys , O. , Corvilain , P. , Aeby , A. , Sculier , C. , Holmes , N. , Brookes , M. , Goldman , S. , Wens , V. , & De Tiège , X. ( 2022 ). On-scalp optically pumped magnetometers versus cryogenic magnetoencephalography for diagnostic evaluation of epilepsy in school-aged children . Radiology , 304 , 429 – 434 . 10.1148/radiol.212453 35503013

[b17] Feys , O. , Corvilain , P. , Van Hecke , A. , Sculier , C. , Rikir , E. , Legros , B. , Nicolas , G. , Leurquin-Sterk , G. , Holmes , N. , Brookes , M. , Goldman , S. , Wens , V. , & De Tiège , X. ( 2023 ). Recording of ictal epileptic activity using on-scalp magnetoencephalography . Annals of Neurology , 93 , 419 – 421 . 10.1002/ana.26562 36480016

[b18] Feys , O. , Ferez , M. , Corvilain , P. , Schuind , S. , Rikir , E. , Legros , B. , Gaspard , N. , Holmes , N. , Brookes , M. , Wens , V. , & De Tiège , X. ( 2024 ). On-scalp magnetoencephalography can detect mesial temporal lobe epileptiform discharges . Annals of Neurology , 95 , 620 – 622 . 10.1002/ana.26844 38050959

[b19] Hamalainen , M. , Hari , R. , Ilmoniemi , R. , Knuutila , J. , & Lounasmaa , O. ( 1993 ). Magnetoencephalography-theory, instrumentation, and applications to noninvasive studies of the working human brain . Reviewed Modern Physics , 65 , 413 – 497 . 10.1103/revmodphys.65.413

[b20] Hill , R. , Boto , E. , Rea , M. , Holmes , N. , Leggett , J. , Coles , L. , Papastavrou , M. , Everton , S. , Hunt , B. , Sims , D. , Osborne , J. , Shah , V. , Bowtell , R. , & Brookes , M. ( 2020 ). Multi-channel whole-head OPM-MEG: Helmet design and a comparison with a conventional system . NeuroImage , 219 , 116995 . 10.1016/j.neuroimage.2020.116995 32480036 PMC8274815

[b21] Hill , R. , Schofield , H. , Boto , E. , Rier , L. , Osborne , J. , Doyle , C. , Worcester , F. , Hayward , T. , Holmes , N. , Bowtell , R. , Shah , V. , & Brookes , M. ( 2024 ). Optimising the sensitivity of optically-pumped magnetometer magnetoencephalography to gamma band electrophysiological activity . Imaging Neuroscience , 2 , 1 – 19 . 10.1162/imag_a_00112 PMC12247564 40800442

[b22] Hillebrand , A. , Holmes , N. , Sijsma , N. , O’Neill , G. , Tierney , T. , Liberton , N. , Stam , A. , van Klink , N. , Stam , C. , Bowtell , R. , Brookes , M. , & Barnes , G. ( 2023 ). Non-invasive measurements of ictal and interictal epileptiform activity using optically pumped magnetometers . Scientific Reports , 13 , 4623 . 10.1038/s41598-023-31111-y 36944674 PMC10030968

[b23] Holmes , N. , Leggett , J. , Boto , E. , Roberts , G. , Hill , R. , Tierney , T. , Shah , V. , Barnes , G. , Brookes , M. , & Bowtell , R. ( 2018 ). A bi-planar coil system for nulling background magnetic fields in scalp mounted magnetoencephalography . NeuroImage , 181 , 760 – 774 . 10.1016/j.neuroimage.2018.07.028 30031934 PMC6150951

[b24] Holmes , N. , Rea , M. , Chalmers , J. , Leggett , J. , Edwards , L. , Nell , P. , Pink , S. , Patel , P. , Wood , J. , Murby , N. , Woolger , D. , Dawson , E. , Mariani , C. , Tierney , T. , Mellor , S. , O’Neill , G. , Boto , E. , Hill , R. , Shah , V. , … Bowtell , R. ( 2022 ). A lightweight magnetically shielded room with active shielding . Scientific Reports , 12 , 13561 . 10.1038/s41598-022-17346-1 35945239 PMC9363499

[b25] Holmes , N. , Tierney , T. , Leggett , J. , Boto , E. , Mellor , S. , Roberts , G. , Hill , R. , Shah , V. , Barnes , G. , Brookes , M. , & Bowtell , R. ( 2019 ). Balanced, bi-planar magnetic field and field gradient coils for field compensation in wearable magnetoencephalography . Scientific Reports , 9 , 14196 . 10.1038/s41598-019-50697-w 31578383 PMC6775070

[b26] Iivanainen , J. , Stenroos , M. , & Parkkonen , L. ( 2017 ). Measuring MEG closer to the brain: Performance of on-scalp sensor arrays . NeuroImage , 147 , 542 – 553 . 10.1016/j.neuroimage.2016.12.048 28007515 PMC5432137

[b27] Mellor , S. , Tierney , T. , O’Neill , G. , Alexander , N. , Seymour , R. , Holmes , N. , Lopez , J. , Hill , R. , Boto , E. , Rea , M. , Roberts , G. , Leggett , J. , Bowtell , R. , Brookes , M. , Maguire , E. , Walker , M. , & Barnes , G. ( 2022 ). Magnetic field mapping and correction for moving OP-MEG . IEEE Transactions on Biomedical Engineering , 69 ( 2 ), 528 – 536 . 10.1109/tbme.2021.3100770 34324421 PMC7612292

[b28] Muthukumaraswamy , S. ( 2013 ). High-frequency brain activity and muscle artifacts in MEG/EEG: A review and recommendations . Frontiers in Human Neuroscience , 7 , 138 . 10.3389/fnhum.2013.00138 23596409 PMC3625857

[b29] Nugent , A. , Andonegui , A. , Holroyd , T. , & Robinson , S. ( 2022 ). On-scalp magnetocorticography with optically pumped magnetometers: Simulated performance in resolving simultaneous sources . Neuroimage: Reports , 2 ( 2 ), 100093 . 10.1016/j.ynirp.2022.100093 35692456 PMC9186482

[b30] Nunez , P. L. ( 2002 ). Electroencephalography (EEG) . In V. Ramachandran (Ed.), Encyclopedia of the human brain (pp. 169 – 179 ). Academic Press . 10.1016/b0-12-227210-2/00128-x

[b31] Oostenveld , R. , Fries , P. , Maris , E. , & Schoffelen , J. ( 2011 ). FieldTrip: Open Source Software for Advanced Analysis of MEG, EEG, and invasive electrophysiological data . Computational Intelligence and Neuroscience , 2011 , 156869 . 10.1155/2011/156869 21253357 PMC3021840

[b32] Pfurtscheller , G. , & Lopes da Silva , F. ( 1999 ). Event-related EEG/MEG synchronization and desynchronization: Basic principles . Clinical Neurophysiology , 110 , 1842 – 1857 . 10.1016/s1388-2457(99)00141-8 10576479

[b33] Rea , M. , Boto , E. , Holmes , N. , Hill , R. , Osborne , J. , Rhodes , N. , Leggett , J. , Rier , L. , Bowtell , R. , Shah , V. , & Brookes , M. ( 2022 ). A 90-channel triaxial magnetoencephalography system using optically pumped magnetometers . Annals of the New York Academy of Sciences , 1517 ( 1 ), 107 – 124 . 10.1111/nyas.14890 36065147 PMC9826099

[b34] Rea , M. , Holmes , N. , Hill , R. , Boto , E. , Leggett , J. , Edwards , L. , Woolger , D. , Dawson , E. , Shah , V. , Osborne , J. , Bowtell , R. , & Brookes , M. ( 2021 ). Precision magnetic field modelling and control for wearable magnetoencephalography . NeuroImage , 241 , 118401 . 10.1016/j.neuroimage.2021.118401 34273527 PMC9248349

[b35] Rhodes , N. , Rea , M. , Boto , E. , Rier , L. , Shah , V. , Hill , R. , Osborne , J. , Doyle , C. , Holmes , N. , Coleman , S. , Mullinger , K. , Bowtell , R. , & Brookes , M. ( 2023 ). Measurement of frontal midline theta oscillations using OPM-MEG . NeuroImage , 271 , 120024 . 10.1016/j.neuroimage.2023.120024 36918138 PMC10465234

[b36] Robinson , S. , Andonegui , A. , Holroyd , T. , Hughes , K. , Alem , O. , Knappe , S. , Maydew , T. , Griesshammer , A. , & Nugent , A. ( 2022 ). Cross-axis dynamic field compensation of optically pumped magnetometer arrays for MEG . NeuroImage , 262 , 119559 . 10.1016/j.neuroimage.2022.119559 35970471 PMC9464713

[b37] Ru , X. , He , K. , Lyu , B. , Li , D. , Xu , W. , Gu , W. , Ma , X. , Liu , J. , Li , C. , Li , T. , Zheng , F. , Yan , X. , Yin , Y. , Duan , H. , Na , S. , Wan , S. , Qin , J. , Sheng , J. , & Gao , J. ( 2022 ). Multimodal neuroimaging with optically pumped magnetometers: A simultaneous MEG-EEG-fNIRS acquisition system . NeuroImage , 259 , 119420 . 10.1016/j.neuroimage.2022.119420 35777634

[b38] Schofield , H. , Boto , E. , Shah , V. , Hill , R. , Osborne , J. , Rea , M. , Doyle , C. , Holmes , N. , Bowtell , R. , Woolger , D. , & Brookes , M. ( 2023 ). Quantum enabled functional neuroimaging: The why and how of magnetoencephalography using optically pumped magnetometers . Contemporary Physics , 63 , 161 – 179 . 10.1080/00107514.2023.2182950 PMC10923587 38463461

[b39] Tierney , T. , Alexander , N. , Mellor , S. , Holmes , N. , Seymour , R. , O’Neill , G. , Maguire , E. , & Barnes , G. ( 2021 ). Modelling optically pumped magnetometer interference in MEG as a spatially homogeneous magnetic field . NeuroImage , 244 , 118484 . 10.1016/j.neuroimage.2021.118484 34418526

[b40] Tierney , T. , Holmes , N. , Mellor , S. , Lopez , J. , Roberts , G. , Hill , R. , Boto , E. , Leggett , J. , Shah , V. , Brookes , M. , Bowtell , R. , & Barnes , G. ( 2019 ). Optically pumped magnetometers: From quantum origins to multi-channel magnetoencephalography . NeuroImage , 199 , 598 – 608 . 10.1016/j.neuroimage.2019.05.063 31141737 PMC6988110

[b41] Vivekananda , U. , Mellor , S. , Tierney , T. , Holmes , N. , Boto , E. , Leggett , J. , Roberts , G. , Hill , R. , Litvak , V. , Brookes , M. , Bowtell , R. , Barnes , G. , & Walker , M. ( 2020 ). Optically pumped magnetoencephalography in epilepsy . Annals of Clinical and Translational Neurology , 7 , 397 – 401 . 10.1002/acn3.50995 32112610 PMC7085997

[b42] Whitham , E. , Pope , K. , Fitsgibbon , S. , Lewis , T. , Clark , C. , Loveless , S. , Broberg , M. , Wallace , A. , DeLosAngeles , D. , Lillie , P. , Hardy , A. , Fronsko , R. , Pulbrook , A. , & Willoughby , J. ( 2007 ). Scalp electrical recording during paralysis: Quantitative evidence that EEG frequencies above 20 Hz are contaminated by EMG . Clinical Neurophysiology , 118 ( 8 ), 1877 – 1888 . 10.1016/j.clinph.2007.04.027 17574912

[b43] Yoshinaga , H. , Nakahori , T. , Ohtsuka , Y. , Oka , E. , Kitamura , Y. , Kiriyama , H. , Kinugasa , K. , Miyamoto , K. , & Hoshida , T. ( 2002 ). Benefit of simultaneous recording of EEG and MEG in dipole localization . Epilepsia , 43 ( 8 ), 924 – 928 . 10.1046/j.1528-1157.2002.42901.x 12181013

