# Supplementary material for: Simultaneous whole-head electrophysiological recordings using EEG and OPM-MEG
Source: Imaging Neurosci (Camb). 2024 May 20;2:imag-2-00179. doi: 10.1162/imag_a_00179 (PMC12247592; doi:10.1162/imag_a_00179)
Supplement: Supplementary Material [file imag_a_00179-supp.pdf]

Simultaneous whole-head electrophysiological recordings using EEG and OPM-MEG

Zelekha A. Seedat, Kelly St Pier, Niall Holmes, Molly Rea, Layla Al-Hilaly, Rosemarie Pardington, Karen J. Mullinger, J. Helen Cross, Elena Boto and Matthew J. Brookes

SUPPLEMENTARY INFORMATION

Additional results

The nature of the EEG signal means that it is recorded relative to a reference electrode. Different montages can be used to view the EEG recording in different ways – for example, referencing to an electrode at the front of the head will amplify signals at the back of the head. In our analyses in the main manuscript, we employed an average reference – in other words, each electrode was measuring activity relative to the average over all electrodes (excluding noisy channels). This makes the EEG data more comparable with OPM-MEG, which measures a value of magnetic field without reference to another location. To ensure that our results were not dependent on the referencing scheme employed, analyses were also repeated with the common reference. The following figures (S1 – S4) show those results.

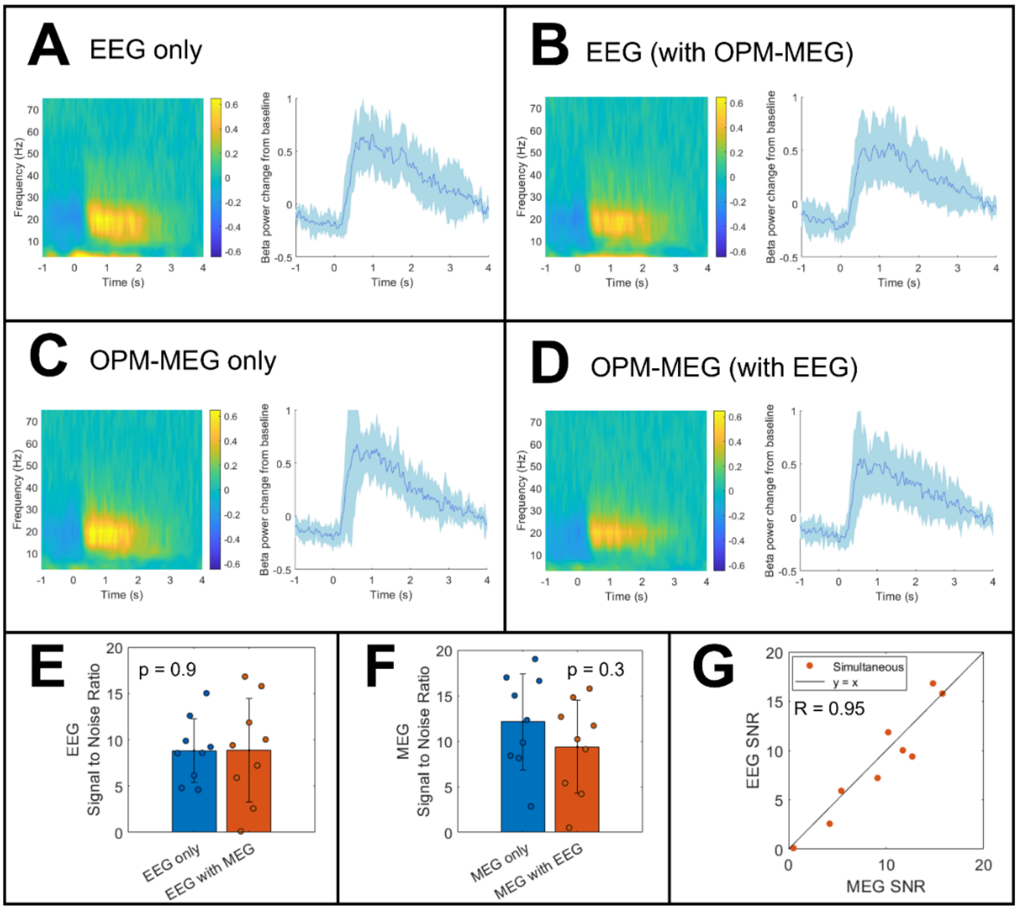

Figure S1: Equivalent to Figure 3 in the main manuscript, but derived using a single common recording reference (Fcz)

For the motor task, the results for the peak channels remain largely unchanged (Figure S1). In other words, the task modulated response (motor related beta desynchronisation (MRBD) followed by a post-movement beta rebound (PMBR)) is clearly visible in both EEG and OPM-MEG measured alone or simultaneously and there is no significant difference in signal-to-noise ratios (SNRs) when simultaneous recordings took place. The difference between reference schemes is minimal: for EEG alone, the SNR was 10 ± 5 (mean ± std) for the average reference, and 9 ± 3 (mean ± std) for the CRR. For EEG in the presence of OPM-MEG the SNR was 9 ± 6 (mean ± std) when either the average reference or the CRR was used.

The choice of referencing scheme had much greater impact on our measure of signal spread (Figure S2). The task-modulated response in EEG appears to be more diffuse when the CRR is used so that the fraction of sensors highly correlated with the peak sensor changes from 0.5 ± 0.3 (mean ± std) for the average reference to 0.8 ± 0.1 (mean ± std) for the CRR. In either case, there is a significantly higher proportion of sensors highly correlated to the peak sensor in EEG than in OPM-MEG.

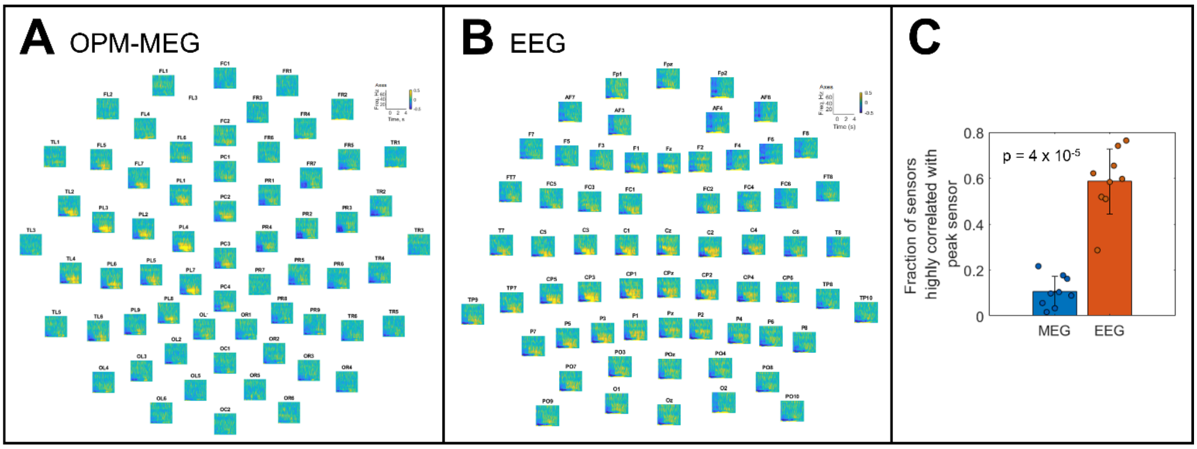

Figure S2: Equivalent to Figure 4 in the main manuscript but generated with a common recording reference (FCz).

For the alpha generation task, traces were again visibly inspected but with the CRR for EEG. Figure S3 shows these results for an example participant. In both EEG and OPM-MEG there is a visible alpha rhythm (panels A and B respectively). Frequency analysis across all channels shows a clear peak at approximately 10Hz, maximal posteriorly for both EEG and OPM-MEG in agreement with our initial results. It is worth noting that there is markedly less alpha visible in EEG frontal channels with this choice of reference in comparison with the average reference (even when posterior channels had been excluded from the average), see Figure S3 and Figure 4 in the main manuscript.

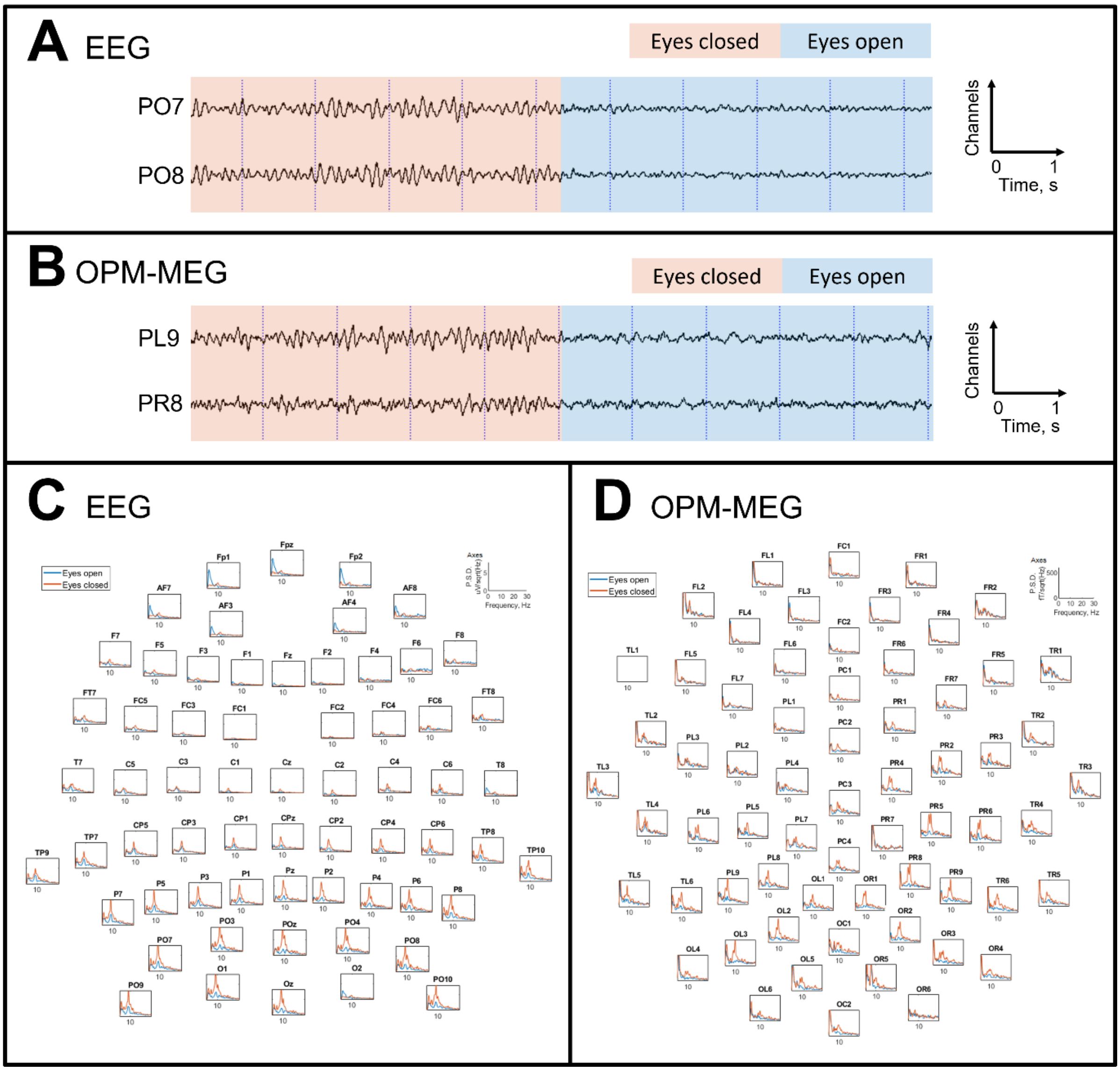

Figure S3: Equivalent to Figure 5 in the main manuscript but generated with a common recording reference (FCz).

For each participant the peak sensor was selected, and power spectral density (PSD) plots were computed and then averaged across all participants. Figure S4 shows these PSDs for both EEG and OPM-MEG, with the common recording reference used for EEG. In all 4 cases (EEG alone, EEG recorded with OPM-MEG, OPM-MEG alone, and OPM-MEG recorded with EEG) there is a prominent alpha peak during the eyes closed period. Panels C and F show the signal contrast between eyes open and eyes closed conditions for EEG and OPM-MEG respectively. In agreement with our initial findings, there is no significant difference in signal contrast between EEG alone (3 ± 1) (mean ± std) and EEG in the presence of OPM-MEG (4 ± 1) (mean ± std), p = 0.8.

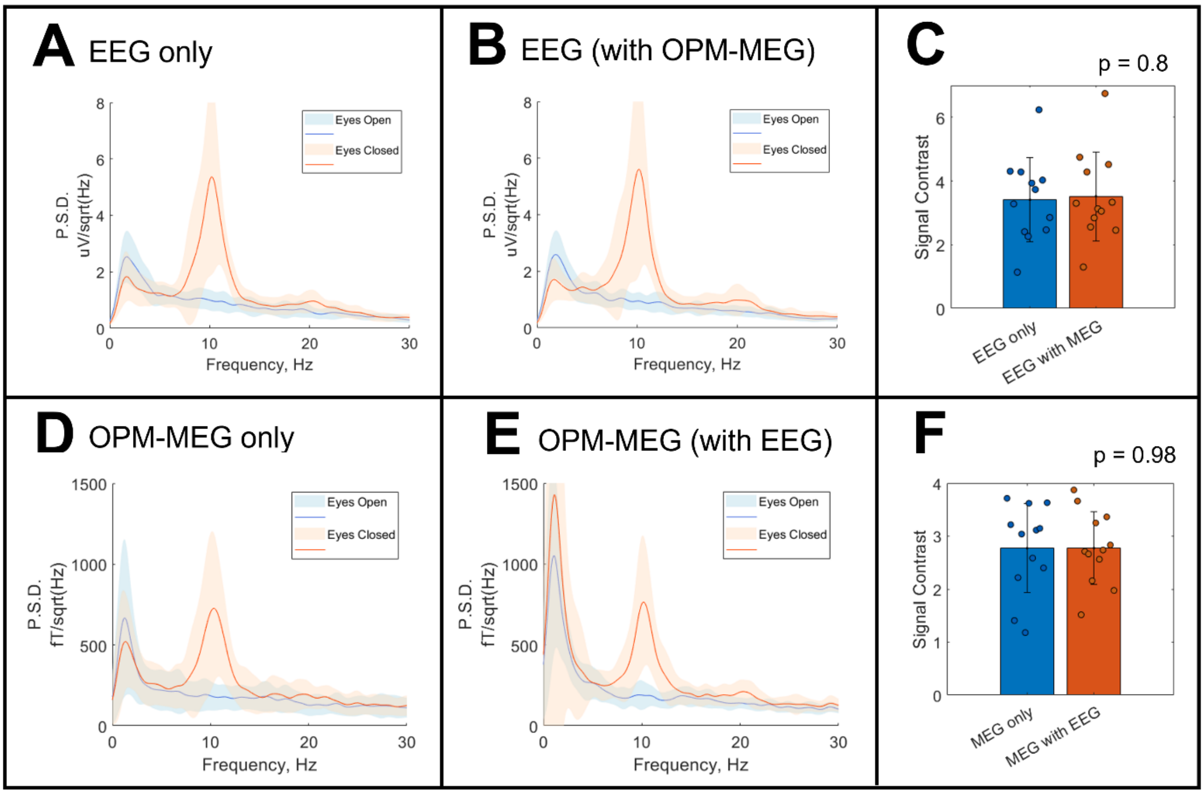

Figure S4: Equivalent to Figure 6 in the main manuscript but generated with a common recording reference (FCz).

Additional methodological detail: Sensor layouts:

Sensor layouts for OPM-MEG and EEG are shown in Figure S5.

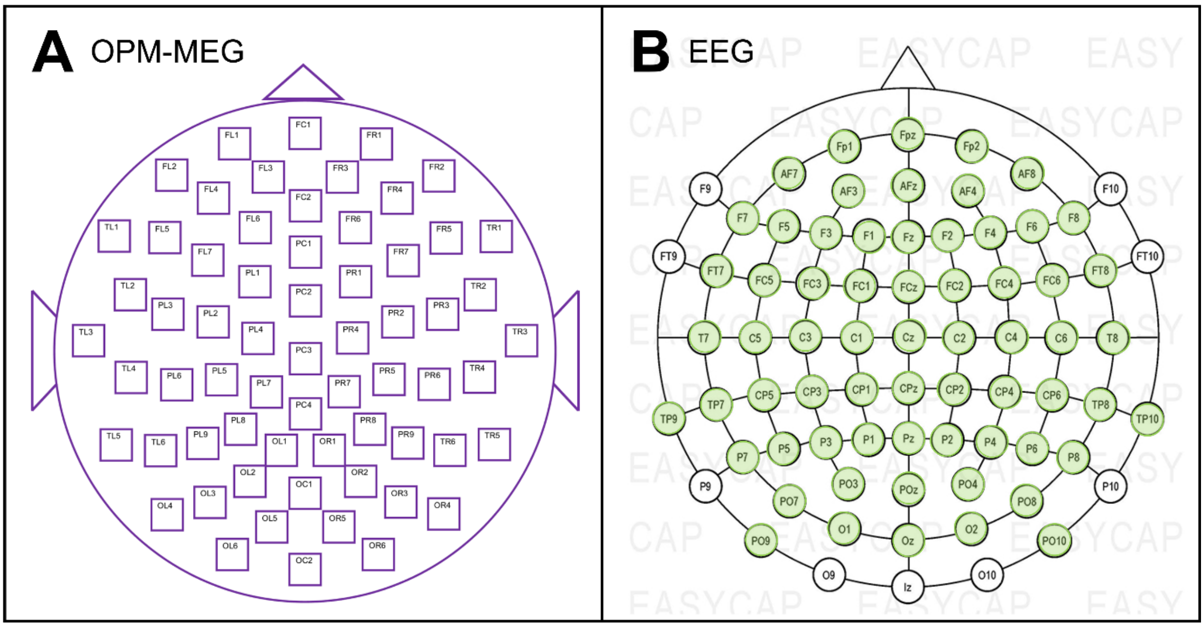

Figure S5: Sensor locations for OPM-MEG and EEG. (A) A dual axis OPM is placed in each of the sensor positions marked out by a square. (B) Electrode locations are dictated by the Brain Products cap used for EEG. A 10-10 layout was followed, and the electrodes highlighted in green were mounted in this cap. AFz was the ground electrode, and FCz was the reference electrode when the common recording reference was used.

Additional methodological detail: task paradigm:

Within each scanning session. Participants performed 5 tasks, but only 2 are reported in this paper (motor task and alpha generation task). The remaining tasks were:

1. A 7-minute resting state recording while participants watched the inscapes video (https://www.headspacestudios.org/inscapes).
2. A continuous visual stimulus with participants watching a reversing checkerboard at a reversal rate of 4Hz (for 25 s).
3. A 1-minute recording of characteristic artefacts often seen in EEG data (yawning, shaking head, ocular artefacts etc.).

Additional methodological detail: Field nulling:

During the field nulling analysis, RMS magnetic field was estimated from the uniform field and field gradient components computed on a spherical surface containing the head and the range of movements as:

$$RMS = \sqrt{B_x^2 + B_y^2 + B_z^2 + \frac{2}{3}(g_1^2 + g_2^2 + g_3^2 + 3g_4^2 + g_5^2)R^2}$$

The radius of the sphere is R; B_x, B_y, and B_z are the uniform magnetic field components; and g_{1-5} are the magnetic field gradients ($g_1 = \frac{dB_z}{dy} = \frac{dB_y}{dz}$, $g_2 = \frac{dB_y}{dx} = \frac{dB_x}{dy}$, $g_3 = \frac{dB_z}{dx} = \frac{dB_x}{dz}$, $g_4 = 2\frac{dB_x}{dx} = -\frac{dB_y}{dy} - \frac{dB_z}{dz}$, $g_5 = \frac{dB_y}{dy} = -\frac{dB_z}{dz}$). See Rea et al. 2021 for more detail.
